# Evaluation of Age and Gender-Related Patterns in Clinical Features and Hematological Findings Among Hypothyroidism Patients in the Al-Jouf Region of Saudi Arabia: A Hospital-Based Study

**DOI:** 10.7759/cureus.56161

**Published:** 2024-03-14

**Authors:** Farooq A Wani, Ashokkumar Thirunavukkarasu, Hatim Alrashed, Abdalrhman S Alblwan, Yazeed M Alfuhigi, Mohammed Dilli, Layth Alruwaili

**Affiliations:** 1 Department of Pathology, Jouf University, Sakaka, SAU; 2 Department of Family and Community Medicine, Jouf University, Sakaka, SAU; 3 Department of Medicine and Surgery, Jouf University, Sakaka, SAU; 4 Department of Medicine and Surgery, Jouf university, Sakaka, SAU

**Keywords:** clinical features, lab findings, co-morbidities, hematological, thyroid hormones, hypothyroidism

## Abstract

Background: The prevalence of hypothyroidism is high in Saudi Arabia and the contributing factors are iodine deficiency and lack of balanced nutrition. This study aims to correlate the gender, age, and presence of co-morbidities with the laboratory findings and clinical presentation.

Methodology: A cross-sectional study was done in the hospitals of the Al-Jouf region. The files of the patients diagnosed with hypothyroidism from the last two years were retrieved by non-probability consecutive sampling technique. IBM SPSS Statistics for Windows, Version 23, (Released 2015; IBM Corp., Armonk, New York, United States) was used for data entry and analysis. Descriptive statistics were presented as frequencies and proportions (for qualitative variables) and mean and standard deviation (SD) (for continuous data). Associated factors were identified through a chi-square test. A p-value less than 0.05 was considered statistically significant.

Results: Most of the patients were females within the age group of 36 to 50 years. Significant differences were observed between male and female patients with respect to the FT4 levels, hemoglobin (Hb) levels, mean corpuscular volume (MCV), mean corpuscular hemoglobin (MCH), mean corpuscular hemoglobin concentration (MCHC) values, and RBC counts (p-values of <0.001, <0.001, <0.001, <0.001, <0.001 and <0.001, respectively). However, no significant differences were observed between male and female patients in the TSH levels and hematocrit values. Most of the patients were euthyroid (77.24%). The pattern of thyroid function status did not show significant differences with respect to the gender of participants and the different age groups (p-values of 0.447 and 0.775, respectively). The most common co-morbidities observed were diabetes and hypertension. No significant association between the co-morbidities and the pattern of thyroid function status was observed. The most common symptoms were epigastric pain, fatigue, constipation, drowsiness, altered bowel habits, and weight gain.

Conclusion: This hospital-based study provides valuable insights into some epidemiological characteristics, clinical features, and hematological findings in hypothyroidism patients of the Al-Jouf region. Significant differences were observed between male and female patients with respect to the FT4 levels, Hb levels, MCV, MCH, MCHC values, and RBC counts. The findings strengthen the existing knowledge base and emphasize the importance of timely detection and management of hypothyroidism in this population. Implementation of salt iodination programs and a timely evaluation of the hematological parameters is recommended. Further research is warranted to delve into the hidden mechanisms and long-term ramifications of hematological changes associated with hypothyroidism.

## Introduction

Thyroid hormones are amongst the most essential hormones in growth, development, and metabolism [1,2]. Their deficiency may give rise to many diseases both in adults and children. Hypothyroidism is a very prominent endocrinal disorder and requires appropriate attention as such, especially since it has noticeably increased in prevalence [3,4]. Thyroid hormones are involved in regulating multiple physiological processes like the regulation of body metabolism, neurological development in children, bone maintenance, and proper functioning of the heart, gastrointestinal tract, and muscles, and influence the functions of other glands. Hence a deficiency of these hormones can have detrimental effects on the body, which include significant impact on various organ systems, leading to a range of clinical manifestations. [5,6].

Many factors contribute to the dysfunction of the thyroid gland, including gender predisposition, nutritional status, genetic susceptibility, iodine deficiency, irradiation, and geographical area. Regular screening tests and laboratory examinations are strongly advised to detect thyroid disease early and initiate appropriate management [7].

The prevalence of hypothyroidism has been reported to be high (47.34%) in Saudi Arabia in a study done by Lamfon, 2008, and the contributing factors were found to be a deficiency of iodine and bad nutrition [8]. Al Shahrani et al., 2016, did a systematic review of the epidemiology of thyroid diseases in the Arab world. They reported that the prevalence of different types of thyroid diseases in the Arab world ranged from 6.18 to 47.34%; the prevalence of hypothyroidism was reported as 6.18% in Libya and 47.34% in Saudi Arabia. They identified that gender, dietary practices, deficiency of iodine, family history, diabetes, and X-rays were risk factors for the different types of thyroid diseases. Many of the risk factors highlighted are potentially modifiable, which emphasizes the importance of public health programs in tackling the disorder [7].

Alruwaili et al., 2018, in a study in Arar City, Saudi Arabia, observed that the prevalence of hypothyroidism was 25.5% and females (57.7%) were more affected than males and 40% of the cases had a positive family history [9]. Aljabri et al., 2019, observed that hypothyroidism was highly prevalent in a cohort of Saudis. They found 29.1% of cases with hypothyroidism, out of which 85.7% cases were female, with a male-to-female ratio of 1:6 [10].

Thyroid hormones significantly affect the hematological parameters including the cell counts and various blood cell indices. These effects are directly mediated by the stimulation of erythrocyte precursors as well as mediated indirectly through increased erythropoietin production [11]. Hypothyroidism results in a variety of anemias which include normocytic, microcytic, and macrocytic types [12-14]. In fact, hypothyroidism has been associated with subnormal levels of hemoglobin, serum iron, and vitamin B12 levels [15]. Serum ferritin and serum folic acid levels are also reduced [16,17].

This study aimed to correlate the significance of gender, age, and various co-morbidities with the laboratory findings and clinical presentation. The methodology that was determined was to collect the data by interpreting patients' files.

## Materials and methods

A cross-sectional study was done in the hospitals of the Al-Jouf region of Saudi Arabia. The study participants were patients diagnosed with hypothyroidism in the last two years. Ethical approval was taken from the Ministry of Health, Research Ethics Committee, Qurayyat Health Affairs via Approval Project No. 2022-21 to obtain information from the patient’s records; hence no patient consent was required. The research involved no risk to both participants and patients. All patient files of the last two years were retrieved after necessary approval; the technique of non-probability consecutive sampling was applied to select the required number of files. All the files were arranged in ascending order according to the hospital registration number and every fourth file was selected.

Data collected included demographical features like age and gender; hematological lab findings including hemoglobin (Hb), hematocrit (HCT), RBC count, mean corpuscular volume (MCV), mean corpuscular hemoglobin (MCH), mean corpuscular hemoglobin concentration (MCHC), and red cell distribution width (RDW); thyroid hormone profile like TSH and FT4 levels; clinical features of hypothyroidism patients; and presence of co-morbidities like hypertension, diabetes, chronic lung disease, chronic renal failure and cancer.

The inclusion criterion was patients diagnosed with hypothyroidism in the past two years attending Al-Jouf region hospitals. The exclusion criteria were patients whose records were missing and patients whose data was not complete.

Statistical analysis

Data entry and analysis were performed using IBM SPSS Statistics for Windows, Version 23, (Released 2015; IBM Corp., Armonk, New York, United States). Descriptive statistics were presented as frequencies and proportions (for qualitative variables) and mean and standard deviation (SD) (for continuous data). Associated factors were identified through a chi-square test. A p-value less than 0.05 was set as a statistically significant value.

## Results

The majority of the patients in our study were females and belonged to the age group of 36 to 50 years (Table 1). 

**Table 1:** Gender and age subgroup of the study population (n = 312) The data has been represented as N, and %.

Age group (years)	Gender		
	Female	Male	Total
Up to 35	82 (77.36%)	24 (22.64%)	106
36 to 50	101 (82.79%)	21 (17.21%)	122
Above 50	62 (73.81%)	22 (26.19%)	84
Total	245 (78.53%)	67 (21.47%)	312

The patient’s thyroid statuses were identified based on TSH and FT4 levels, as the following: A. Primary hypothyroidism: serum TSH > 4.94 IU/mL and FT4 < 0.7 ng/dL. B. Subclinical hypothyroidism: normal FT4 (0.85-1.4 ng/dL) and serum TSH higher than the upper limit of reference range >4.94 IU/mL. C. Euthyroid: normal FT4 and TSH.

**Figure 1:**
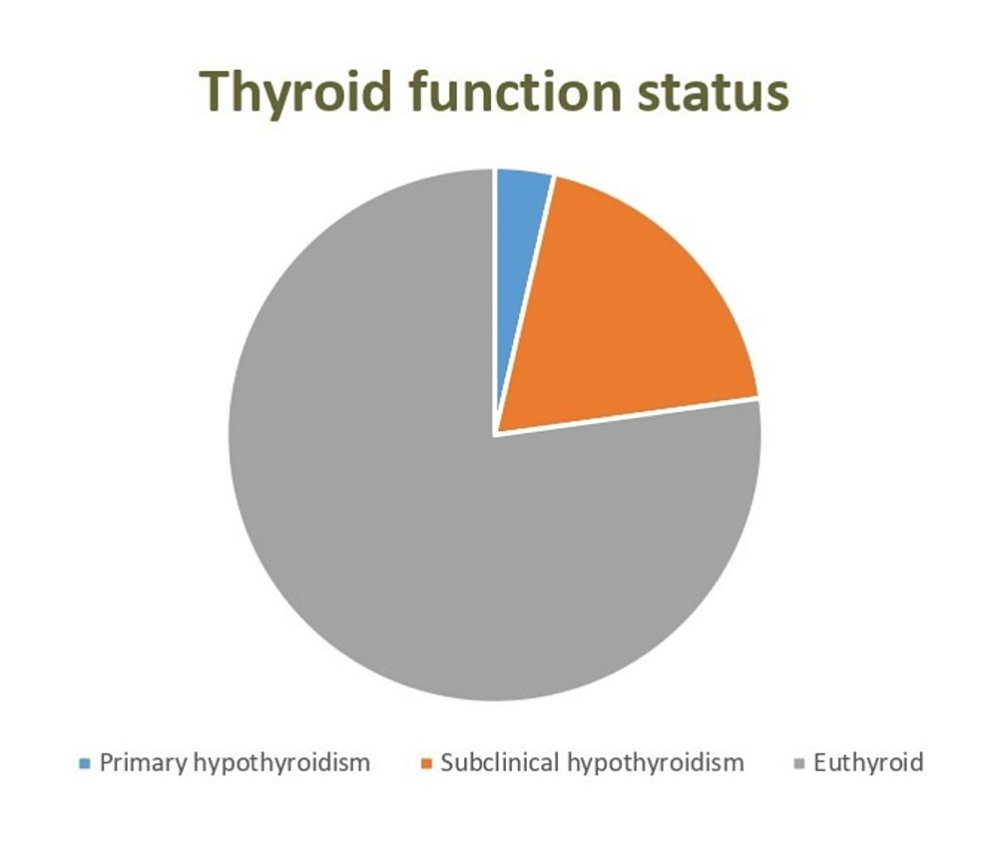
Thyroid function status among the studied patients (n=312)

The majority of patients were euthyroid (241, 77.24%), followed by patients with subclinical hypothyroidism (60, 19.23%) and primary hypothyroidism (11, 3.52%) (Figure 1).

Significant differences were observed between male and female patients with respect to the FT4 levels, Hb levels, MCV, MCH, MCHC values, and RBC counts. However, no significant differences were observed between male and female patients in the TSH levels and HCT (Table 2).

**Table 2:** Characteristics of the study population stratified by gender (n=312). The data has been represented as Mean±SD. A p-value <0.05 is considered significant. Hb: hemoglobin; HCT: hematocrit; MCV: mean corpuscular volume; MCH: mean corpuscular hemoglobin; MCHC: mean corpuscular hemoglobin concentration

Parameter	Female		Male		p-value*
	Mean ± SD	Median ± IQR	Mean ± SD	Median ± IQR	0.444
TSH (µIU/mL)	5.31 ± 11.40	2.70 ± 2.74	2.99 ± 2.13	2.38 ± 2.12	0.406
FT4 (g/dL)	16.52 ± 4.42	16.00 ± 4.40	16.99 ± 4.91	16.50 ± 4.80	<0.001
Hb (g/dL)	12.43 ± 1.62	12.60 ± 1.88	14.69 ± 1.79	15.00 ± 2.00	<0.001
HCT (%)	38.68 ± 5.07	39.00 ± 5.65	44.02 ± 6.69	45.00 ± 7.30	0.088
MCV (fL)	82.01 ± 9.14	83.00 ± 10.17	83.26 ± 11.00	84.00 ± 9.60	<0.001
MCH (Pg)	26.97 ± 4.45	27.00 ± 4.00	28.00 ± 3.04	28.00 ± 2.60	<0.001
MCHC (g/dL)	32.03 ± 2.01	32.00 ± 2.20	32.99 ± 1.99	33.00 ± 2.00	<0.001
RBC (10^12^/L)	4.68 ± 0.57	4.70 ± 0.76	5.19 ± 0.71	5.14 ± 0.84	<0.001

Most of the patients were euthyroid followed by sub-clinical hypothyroidism and primary hypothyroidism patients. However, the pattern of thyroid function status did not show significant differences with respect to the gender of participants and the different age groups (Table 3).

**Table 3:** Pattern of thyroid function status of the patients in different genders and age subgroups (n=312). The data has been represented as N and %. A p-value <0.05 is considered significant.

Gender		Primary hypothyroidism n (%)	Subclinical hypothyroidism n (%)	Euthyroid n (%)	p-value
	Male	1 (1.5)	11 (16.4)	55 (82.1)	0.447
	Female	10 (4.1)	49 (20)	186 (75.9)	
Age group	Up to 35	2 (1.9)	20 (18.9)	84 (79.2)	0.775
	36 to 50	5 (4.1)	22 (18)	95 (77.9)	
	More than 50	4 (4.8)	18 (21.4)	62 (73.8)	

Table 4 depicts the correlation between thyroid hormones and blood parameters. Serum T3 was significantly correlated with RBC counts (0.183/0.004), HCT (0.213/0.001), and Hb (0.194/0.003), and serum T4 was not significantly correlated with any of the evaluated blood parameters. Regarding serum TSH, we found a significant correlation with all parameters except HCT and MCHC.

**Table 4:** Correlation between thyroid hormones and hematological parameters. The data presented here is Spearman’s rho/p-value. Hb: hemoglobin; HCT: hematocrit; MCV: mean corpuscular volume; MCH: mean corpuscular hemoglobin; MCHC: mean corpuscular hemoglobin concentration

Parameters	T3	T4	TSH
RBC	0.183/0.004	-0.050/0.320	0.651/<0.001
HCT	0.213/0.001	0.095.0.061	-0.037/0.462
Hb	0.194/0.003	0.060/0/238	0.634/<0.001
WBC	0.058/0.262	0.053/0.297	0.180/0.001
MCV	-0.003/0.957	0.046/0.360	0.208/<0.001
MCH	-0.001/0.988	-0.022/0.662	-0.202/0.001
MCHC	0.082/0.112	-0.016/0.753	0.045/0.367
Platelets	0.095/0.063	0.072/0.153	0.134/0.008

The most common co-morbidities observed in our study were diabetes and hypertension. No significant association between the various co-morbidities and the pattern of thyroid function status was observed (Table 5). 

**Table 5:** Pattern of thyroid function status of the patients and the presence of chronic diseases (n=312). The data has been represented as N and %. A p-value <0.05 is considered significant. CLD: chronic liver disease; CRF: chronic renal failure

		Primary hypothyroidism n (%)	Subclinical hypothyroidism n (%)	Euthyroid n (%)	p-value
Diabetes mellitus	Yes	5 (7)	14 (19.7)	52(73.2)	0.180
	No	6 (2.5)	46 (19.1)	189(78.4)	
Hypertension	Yes	5 (6.9)	10 (13.9)	57 (79.2)	0.303
	No	6 (2.5)	50 (20.9)	183 (79.2)	
CLD	Yes	1 (14.3)	1 (14.3)	5 (71.4)	0.290
	No	10 (3.8)	59 (18.3)	236 (77.4)	
CRF	Yes	0 (0)	7 (33.3)	14 (66.7)	0.406
	No	11 (3.8)	53 (18.3)	225 (77.9)	
Cancer	Yes	1 (16.7)	2 (33.3)	3 (50)	0.121
	No	10 (3.3)	58 (19)	238 (77.8)	

A range of clinical manifestations were observed ranging from subtle symptoms like fatigue to frank manifestations like depression and cognitive dysfunction. The most common symptoms observed were epigastric pain, fatigue, constipation, drowsiness, altered bowel habits, and weight gain (Table 6).

**Table 6:** Pattern of clinical symptoms among the studied patients (n=312). The data has been represented as N and %.

Symptoms	Frequency (n)	Proportion (%)
Fatigue	99	31.7
Weight gain	52	16.7
Puffy face	15	4.8
Hoarseness	19	6.1
Muscle weakness	16	5.1
Edema	23	7.4
Menstrual disturbances	24	7.7
Hair loss	22	7.1
Hearing impairment	2	0.6
Depression	3	1
Altered bowel habits	53	17
Dry eyes	49	15.7
Paresthesia	14	4.5
Cognitive dysfunction	7	2.2
Drowsiness	53	17
Epigastric pain	103	33
Constipation	72	23.1

## Discussion

Demographic distribution

The findings from this hospital-based study highlight several key aspects that confer an extensive understanding of the disease in this specific population. The study population comprised predominantly females, with the majority falling within the age group of 36 to 50 years. This distribution aligns with the generally higher prevalence of hypothyroidism in females and an increased incidence observed with advancing age. Predominance of females and similar age patterns has been observed in other studies done by Alruwaili et al., 2018; Aljabri et al., 2019 and Alqahtani, 2021 [9,10,18]. However, in a study done by Senthilkumaran et al, 2015, they found that the majority of female participants were between the ages of 55 and 67 years [19].

Thyroid function status

The analysis revealed that a significant proportion of the patients were euthyroid (241, 77.24%), followed by patients with subclinical hypothyroidism and primary hypothyroidism. This distribution is in accordance with the global pattern of thyroid disorders, which underscores the importance of routine thyroid function screening for early identification and management of cases. In a retrospective study done on 600 patients by Bashir et al., 2012, the majority were females and belonged to the euthyroid group [20]. In our study, 60 (19.23%) of patients had sub-clinical hypothyroidism, whereas in a study done by López et al., 2017, 75.2% of the patients had sub-clinical hypothyroidism [21].

Gender and age differences with respect to thyroid function status

The analysis revealed that the pattern of thyroid function status did not show significant differences with respect to the gender and different age groups.

Gender differences with respect to hematological profile

The process of hematopoiesis is hampered in hypothyroid patients as it decreases the proliferative potential of erythroid precursor in the bone marrow [22,23]. Furthermore, the thyroid hormone status determines the expression of thyroid hormone receptors on hematopoietic cells, thereby affecting clonogenicity and promoting apoptosis in CD34+ progenitor cells [24]. Hypothyroidism affects the process of erythropoiesis by inhibiting the release of erythropoietin from the kidney as it decreases gene expression [25,26].

Significant gender-based differences were observed in thyroid hormone levels and hematological parameters. Females exhibited lower FT4 levels, lower Hb levels, smaller MCV, lower MCH, lower MCHC, and lower RBC counts compared to males. These differences may be attributed to hormonal variations between genders, necessitating gender-specific considerations in the management of hypothyroidism. Women are more likely to experience symptoms such as menstrual irregularities, whereas men may show signs such as low libido and erectile dysfunction. Healthcare providers should consider these gender-specific symptoms when evaluating and treating patients. Dosage adjustments may be required as there are hormonal fluctuations during menstrual cycles and especially during pregnancy [27]. Potential drug interactions must be considered especially while treating post-menopausal women on hormone replacement therapies [28].

Alqahtani, 2021, observed a statistically significant difference between the male and female populations for parameters like Hb, HCT, MCV, MCH, MCHC, RDW, and RBC counts. However, he failed to find any significant difference with respect to FT4 and TSH levels [18]. Refaat, 2015, in her study on non‑pregnant Saudi females, observed that hypothyroid females had decreased RBC counts, Hb levels, HCT levels, and deranged iron parameters. Anemia was observed in 44% of cases which included both normocytic normochromic anemia and microcytic hypochromic anemia [22]. Ahmed and Mohammad in their study which included control, hypothyroidism, and hyperthyroidism groups observed significant differences in the RBC, HB, MCV, MCHC, RDW, and WBC counts, whereas platelets showed no significant correlation [11]. Mahanta et al., 2017, observed anemia in about 60% of the cases with 8% having severe anemia, and 52% with mild to moderate anemia [29].

Correlation between thyroid hormones and hematological parameters

Correlation between thyroid hormones and blood parameters revealed a significant correlation of the serum T3 levels with the RBC counts, HCT, and Hb levels, whereas serum T4 was not significantly correlated with any of the evaluated blood parameters. Serum TSH was significantly correlated with all the hematological parameters except HCT and MCHC. Ahmed and Mohammad observed significant differences in the Hb, RBC counts, MCV, MCHC, RDW, and WBC counts, whereas platelets showed no significant correlation [11]. T and Papaiah, 2023, in a retrospective study observed statistically significant differences in TLC, MCHC, and RDW among hypothyroid, hyperthyroid euthyroid, and subclinical hypothyroid respectively. However, they did not observe a significant correlation in Hb, PLT, RBC, PCV, MCV, and MCH among the various thyroid groups [30]. The observed association between hypothyroidism and various red cell parameters in our study highlights the influence of thyroid hormones on RBC production and metabolism, thereby aiding in the development of targeted interventions for improved patient outcomes. The dosage and type of thyroid hormone replacement may be fine-tuned based on the specific populations of patients like iron deficiency anemia and chronic renal failure patients, which can lead to better normalization of RBC parameters [31].

Co-morbidities and pattern of thyroid function status

The study explored the association between thyroid function status and the presence of various co-morbidities like diabetes mellitus, hypertension, chronic liver disease (CLD), chronic renal failure (CRF), and cancer. The most common co-morbidities observed in our study were diabetes and hypertension. Anandhasayanam et al., 2015, observed in their study on hypothyroid patients on levothyroxine treatment that the most common comorbidities were obesity, diabetes, menstrual disturbances, and hypertension [32]. While certain numerical differences were found, we did not find a statistically significant association between the co-morbidities and the pattern of thyroid function status. This advocates that the presence of these co-morbidities may not be a major determinant of thyroid dysfunction in this population.

Clinical symptoms

A range of manifestations associated with hypothyroidism were observed with epigastric pain, fatigue, constipation, drowsiness, altered bowel habits, and weight gain being the most prevalent. This aligns with the classical clinical features of hypothyroidism and underscores the importance of recognizing these clinical features for prompt intervention. In a study done by Mahanta et al., 2017, the most common presenting clinical features were weakness (98%), followed by lethargy (95%), and dry and coarse skin (87%) [29]. Anandhasayanam et al., 2015, observed the most common symptoms were epigastric pain, fatigue, constipation, drowsiness, altered bowel habits, and weight gain [32]. Paudel et al., 2014, in their study on hypothyroidism patients who were on hemodialysis, found that many clinical features such as constipation, cold intolerance, muscular pains, dryness of skin, tingling sensation, periorbital edema, effusions in pericardial, and pleural cavities were significantly observed more in the hypothyroid group as compared to the euthyroid group [33].

The limitations of the study were that it was a single-center study, and we evaluated only age, gender, and co-morbidities as epidemiological characteristics as the data regarding other epidemiological factors was incomplete.

## Conclusions

In conclusion, the study has provided precious insights into the profile of hypothyroidism in the study population. The presence of hypothyroidism appears to be notable, thereby emphasizing the need to have heightened awareness for early detection and timely management of this lesion. Our findings highlighted the myriad clinical manifestations linked with hypothyroidism, which range from insidious symptoms to more obvious signs. This diversity in clinical manifestations highlights the necessity to adopt a comprehensive approach to diagnose and treat such cases.

Moreover, the hematological facets investigated in the study have thrown light on the potential associations between hypothyroidism and certain hematological parameters. The alterations observed in hematological indices may serve as alternative indicators for healthcare professionals while evaluating and managing such patients. We observed significant differences between male and female patients with respect to the FT4 levels, Hb levels, MCV, MCH, MCHC values, and RBC counts. However, the pattern of thyroid function status did not show significant differences with respect to the gender of participants and the different age groups as well as with the various co-morbidities.

We recommend continuing research and joint efforts to improve our comprehension of hypothyroidism, thereby optimizing patient care in the community and beyond. It is recommended to have salt iodination programs and timely evaluation of the hematological parameters with the institution of proper treatment protocols. Furthermore, a multicentric prospective study is recommended to find the association with other epidemiological factors.
